# Transversus Thoracic Muscle Plane Block for Attenuating the Haemodynamic Response to Median Sternotomy: A Case Series

**DOI:** 10.5152/TJAR.2022.21196

**Published:** 2022-12-01

**Authors:** Ashish Walian, Rohan Magoon, Iti Shri, Ramesh Chand Kashav

**Affiliations:** 1 Department of Cardiac Anaesthesia, Atal Bihari Vajpayee Institute of Medical Sciences, Dr. Ram Manohar Lohia Hospital, New Delhi, India

**Keywords:** Cardiac surgical procedures, fascial plane blocks, median sternotomy, multimodal-analgesia, pain, transversus thoracic muscle plane block

## Abstract

With the emergence of opioid-sparing and enhanced recovery pathways, cardiac anaesthesiologists are highly motivated to formulate regional analgesia-centric multimodal regimes, particularly prompted by the inclusion of safer fascial plane blocks to the analgesic repertoire. Ahead of the encouraging literature on perioperative pain relief with the thoracic fascial plane blocks, the fraternity continues to search for promising options for ensuring sternal analgesia. While the novel transversus thoracic muscle plane block emerges as the recent kid on the block for effective sternal analgesia (in the most anatomical sense of the matter), the sporadic case reports and feasibility studies primarily focus on an overall perioperative analgesic role of the block. The index case series describes a noteworthy experience with a pre-induction transversus thoracic muscle plane block administration for attenuating the intraoperative (particularly, median sternotomy) haemodynamic response in adult cardiac surgical patients, with a potential to translate into reduced perioperative fentanyl requirement, augmented recovery, and fast-tracking.

**Main Points**

- Pain following cardiac surgery is multifactorial with median sternotomy being a significant contributor to it.
- Previous studies have demonstrated a role of Transversus Thoracic Muscle Plane Block (TTPB) in decreasing pain following cardiac surgery alongside a reduction in perioperative opioid dependence in cardiac surgery.
- The index case series is a novel attempt where in a preanaesthetic TTPB in 15 consecutive homogenous category of patients, attenuated haemodynamic response to median sternotomy in the anatomico-clinical context with eventual positive effect on postoperative pain scores and augmented recovery following cardiac surgery.

## Introduction

While one cannot condescend to the role of opioids in the conduct of cardiac surgery, the paradigm shift towards opioid-sparing anaesthesia continues to be fuelled by the peculiar adverse effect and addiction profile of opioids. At the same time, the embracement of enhanced recovery pathways further motivates opioid-sparing, resulting in a recent interest in the safer fascial plane blocks for post-cardiotomy pain management.^1-4^

The literature is replete with the mechanisms of pain after cardiothoracic surgery with median sternotomy pain emerging as one of the important contributors.^5-7^ In this context, a recently described transversus thoracic muscle plane block (TTPB) has captivated attention for its potential role in ensuring perioperative sternal analgesia.^6,8-11^ While previous observations employing a TTPB have focused on postoperative pain scores and the use of rescue analgesics,^6,8-11^ the present case series describes the effect of a pre-induction TTPB on the intraoperative haemodynamic response, particularly, to median sternotomy. The perioperative fentanyl requirement, postoperative pain scores, and the recovery profile of the included patients have also been outlined.

## Case Presentation

The case series included 15 consecutive patients aged 18-50 years, American Society of Anaesthesiologists (ASA) physical status II-III who received a planned pre-induction TTPB block prior to undergoing an elective cardiac surgery requiring median sternotomy in the time period from September 2020 to November 2020. The patients undergoing coronary artery bypass grafting, history of systemic hypertension, pre-existing renal and hepatic failure, uncontrolled diabetes mellitus, local anaesthetic (LA) allergy, coagulopathy, psychiatric illness, chronic opioid use, and those who did not consent were excluded.

**Figure 1.**
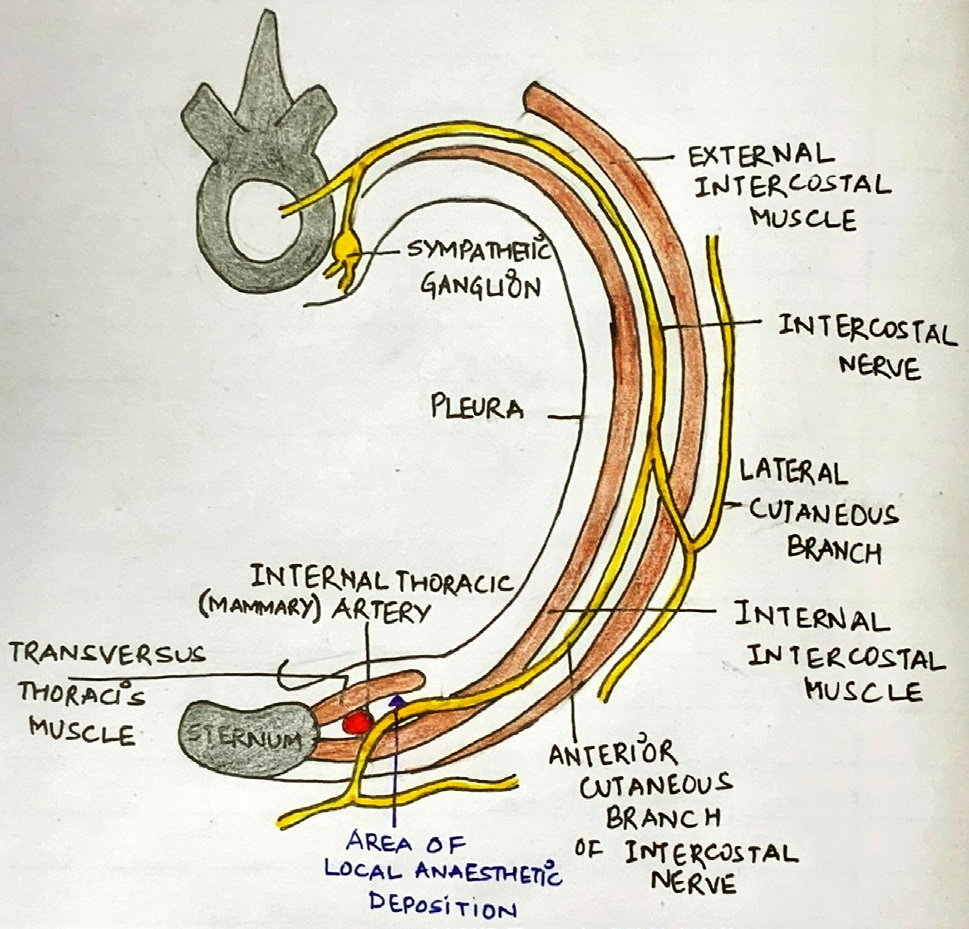
Sketch diagram illustrating the transversus thoracic muscle plane anatomy.

**Figure 2.**
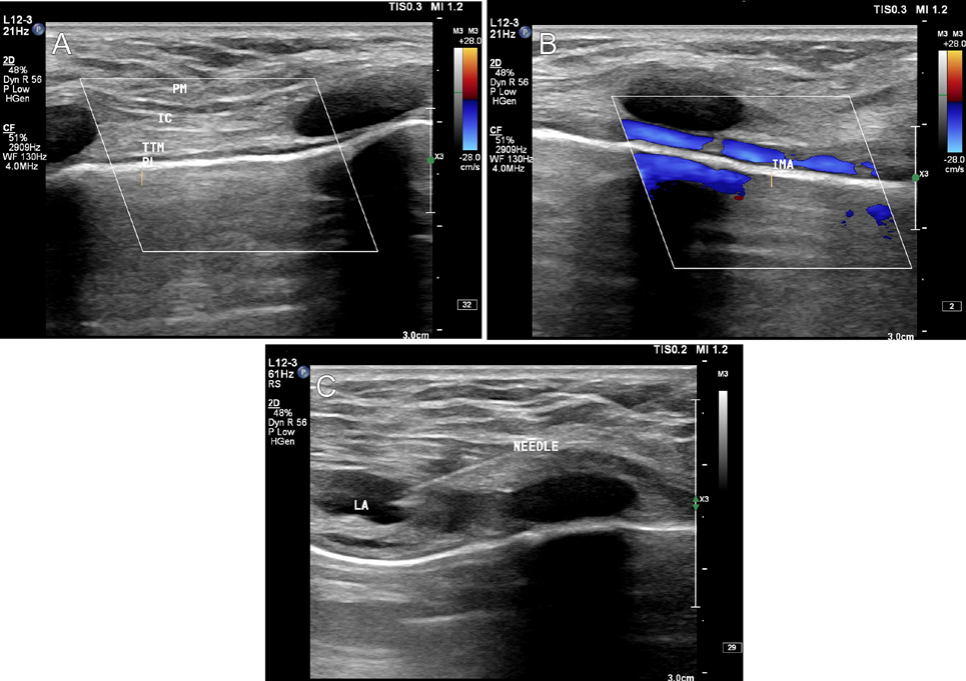
(A) Ultrasonographic depiction of the pectoralis major (PM), internal intercostals (IC), transversus thoracic muscle (TTM), and the pleura (PL). (B) The internal mammary artery (IMA) visualised running between the internal intercostal and transversus thoracic muscle. (C) The in-plane needle tract and tip can be visualised with the local anaesthesia (LA) being deposited in the transversus thoracic muscle plane, pushing the pleura down consequently.

We followed a standard institutional preoperative assessment protocol. In addition, the patients were explained about the Numeric Rating Scale (NRS) for pain assessment (dynamic [on coughing] and static component) while obtaining consent for the block. In the operation theatre, after achieving a Ramsay Sedation Score of 2-3 (employing injection fentanyl 1 µg kg^−1^ and midazolam 0.02 mg kg^−1^), an ultrasonography (USG)-guided TTPB was performed using a high frequency (3-12 MHz) linear transducer (L12-3, Philips Healthcare EPIQ 7 USG machine, Bothell, Wash, USA) after infiltrating the skin with LA. The probe was placed in a parasagittal fashion just lateral to the lateral border of sternum over the third and fourth ribs. The intercostal muscles that lie between the ribs and transversus thoracic muscle (TTM) and the underlying pleura were identified. Herein, the transversus thoracis plane (TTP) that can be found between the internal intercostal muscle and the TTM classifies as the target site for the TTPB (Figures 1).^5,7-10^ After identifying the internal mammary artery and vein (by colour Doppler technique) that run between the intercostals and TTM (Figure 2A and 2B), the probe was moved slightly lateral and the needle was inserted through cephalad-to-caudad using an in-plane approach. While continuously identifying the needle tract, its tip was positioned in the TTP (Figure 2C). After a real-time USG-guided hydrodissection to avoid penetration into the pleura, 30 mL of 0.2% ropivacaine was injected bilaterally in small portions after negative aspiration of blood. The LA spread was confirmed between the costal cartilage and TTM from the T2-T6 intercostal space.

Following the block, general anaesthesia was induced using intravenous doses of 2-3 µg kg^−1^ fentanyl and 0.2-0.3 mg kg^−1^ etomidate, and the trachea was intubated 3 minutes after a neuromuscular blocking dose of rocuronium of 1.0 mg kg^−1^. Anaesthesia was maintained with supplemental doses of midazolam and rocuronium, and the patients were ventilated with a mixture of oxygen, air, and isoflurane to achieve a 0.5 fractional-inspired oxygen concentration and 1.0 minimum-alveolar concentration. A 4 mg kg^−1^ dose of heparin was intravenously administered to achieve an activated clotting time (ACT) > 480 seconds. The cardiopulmonary bypass (CPB) was instituted and maintained under standard institutional protocol. 2 µg kg^−1^ of fentanyl was administered while going-on and coming-off CPB along with the supplemental rocuronium and midazolam boluses. The anaesthetic depth was monitored intraoperatively with a target bispectral index value of 40-60. Subsequent to CPB weaning, the heparin was adequately reversed with protamine in order to achieve the baseline ACT. After surgery, the intubated patients were shifted to cardiac intensive care unit (ICU) and weaned from mechanical ventilation subsequently.

The heart rate (HR) and radial artery transduced systolic, diastolic, and mean arterial pressure (MAP) were monitored. Additional intraoperative fentanyl of 1.0 µg kg^−1^ was administered when the haemodynamic parameters (MAP and HR) demonstrated a rise of ≥20% above the baseline. Postoperatively pain (both static as well as dynamic components) was assessed using NRS scale at extubation, and then at 1 hour, 6 hour, 12 hours and 24 hours following extubation. Other parameters recorded were postoperative fentanyl rescue analgesia (0.5 kg^−1^), postoperative nausea and vomiting (PONV) were recorded. All the patients received intravenous paracetamol of 15 mg kg^−1^ every 8 postoperative hours.

Out of the 15 patients, 3 demonstrated ≥20% increase in MAP and HR at skin incision (Ti) and 4 at median sternotomy (Ts), necessitating an intraoperative fentanyl supplementation. None of the patients manifested a significant haemodynamic response at intubation (Tt) (Table 1) or at any other point during the surgery. All the patients shifted to intensive care unit (ICU) could be extubated within 4-6 hours once the extubation criteria were met. The extubation was uneventful, 4 patients required rescue fentanyl at extubation, and 3 and 5 patients after 12 hours and 24 hours post-extubation, respectively. The mean post-extubation static and dynamic NRS was 2.4 and 3.5, respectively (Table 2). The mean intraoperative fentanyl consumption was 7.93 ± 1.16 µg kg^−1^ which is less compared to the usual tune of 15-20 µg kg^−1^ of fentanyl consumed intraoperatively in cardiac cases at our institute. The mean postoperative fentanyl requirement was 0.5 ± 0.627 µg kg^−1^ and the patients could be fast-tracked (extubation within 6 hours of ICU admission)^12^ with none complaining of PONV.

## Discussion

The elucidation of an attenuated median sternotomy haemodynamic response in the index case series is noteworthy and promising with regards to the sternal analgesic role of TTPB with the potential to translate into reduced perioperative fentanyl requirement, enhanced recovery, and fast-tracking.

While pain after cardiac surgery is essentially multifactorial, median sternotomy classifies as a major contributing factor. Inadequate pain management can aggravate the stress response with potential haemodynamic, metabolic, immunologic, and deleterious organ-function consequences. Sternotomy pain, in particular, interferes with breathing, pulmonary toileting, and ambulation and can culminate as chronic post-sternotomy pain in 17%-88%.^5-7^

Transversus thoracic muscle plane block has captivated interest for a role in ensuring effective sternal analgesia as first described by Ueshima et al.^8^ Speaking strictly anatomically, the chest wall fascial plane blocks cannot truly provide sternal analgesia. However, TTPB can take care of the notorious sternotomy pain by anaesthetising the anterior branches of T2-T6 intercostal nerves.^5,6,11,13^ Moreover, it does not interfere with coagulation management and can be used both in adults and paediatrics. The documented complications include pneumothorax, haemothorax, pericardial puncture, and intravascular injection, and this mandates a meticulous approach.^6,8-11^ We did not encounter any block-related complications.

Fujii et al^6^ TTPB feasibility study in adult cardiac surgery and Zhang et al^11^ application of TTPB for postoperative analgesia in paediatric cardiac surgery constitute encouraging research endeavours in this area. However, the present case series is unique in the context of a pre-induction TTPB administration centralising the focus on intraoperative (particularly, median sternotomy) haemodynamic response alongside pain scores and analgesic requirements.

While both TTPB and parasternal intercostal nerve block have been utilised in cardiac surgical settings, the classical description outlines the plane between the internal intercostal muscles and TTM as the site of LA deposition for TTPB, whereas the latter targets the plane between the external intercostal and pectoralis major muscles.^14^ Talking of LA doses, we employed 30 mL of 0.2% ropivacaine as opposed to Fujii et al^6^ who employed 20 mL of 0.3%-0.5% ropivacaine. Nevertheless, the body weight-adjusted drug dosage was well within the permissible limits.

## Conclusion

The case series suggests that TTPB can mitigate the haemodynamic perturbations at median sternotomy and can be an effective tool for ensuring sternal analgesia. Future randomised controlled trials with a larger sample size are warranted to validate the encouraging findings, in order to explore the potential of individualised procedure-specific analgesic management.^15^

## Figures and Tables

**Table 1.** The Demographics of the Included Patients and the Haemodynamic Parameters at the Baseline (T0), at Intubation (Tt), Skin Incision (Ti), and Median Sternotomy (Ts)

Case	Age/Sex	Surgery	Surgical Duration (Minutes)	CPB Duration (Minutes)	Baseline (T_0_)		Intubation (Tt)		Skin Incision (Ti)		Sternotomy (Ts)	
					SBP/DBP (MAP)	HR	SBP/DBP (MAP)	HR	SBP/DBP (MAP)	HR	SBP/DBP (MAP)	HR
1	36/F	ASD closure	150	35	99/59 (74)	62	111/64 (81)	72	107/62 (78)	70	106/62 (76)	77
2	45/F	ASD closure	145	33	95/57 (71)	81	101/60 (75)	85	98/60 (72)	80	103/61 (76)	81
3	40/M	AVR	180	40	120/63 (84)	96	127/64 (85)	97	129/66 (88)	99	160/77 (107)^*^	119^*^
4	54/M	MVR	195	42	82/50 (62)	79	90/52 (66)	80	91/53 (67)	80	103/63 (75)	86
5	50/F	MVR	210	50	94/55 (69)	78	101/60 (75)	85	104/60 (75)	80	130/65 (85)^*^	104^*^
6	31/F	ASD closure	170	35	91/51 (68)	73	98/57 (71)	77	99/59 (73)	79	103/65(75)	82
7	34/F	ASD closure	160	34	115/57 (74)	95	116/58 (75)	97	120/57 (77)	98	123/60 (79)	99
8	46/M	ASD closure	175	35	117/66 (86)	83	119/67 (88)	85	120/68 (89)	89	130/76 (97)	98
9	44/F	MVR	190	42	95/55 (69)	78	101/61 (76)	85	120/62 (80)^*^	99^*^	135/72 (94)^*^	110^*^
10	54/M	AVR	190	46	100/59 (73)	80	106/63 (77)	83	107/61 (76)	82	111/64 (81)	88
11	48/M	AVR	210	54	102/60 (75)	85	107/64 (78)	82	127/69 (85)^*^	99^*^	111/57 (75)	67
12	40/M	MVR	205	51	93/56 (69)	78	100/61 (75)	85	105/61 (77)	87	107/62 (78)	90
13	46/F	MVR	190	47	114/57 (73)	95	116/60 (76)	97	122/61 (80)	97	128/69 (83)	101
14	50/M	AVR	220	52	107/62 (76)	79	118/61 (80)	93	143/76 (100)^*^	100^*^	150/80 (105)^*^	104^*^
15	52/F	MVR	200	49	119/58 (77)	97	121/63 (81)	95	124/65 (82)	96	129/71 (86)	99

^*^An intraoperative increase in MAP or HR more than 20% was recorded and supplemented with 1 µg kg^−1^ fentanyl.

ASD, atrial septal defect; AVR, aortic valve replacement; F, female; M, male; MVR, mitral valve replacement; SBP, systolic blood pressure; DBP, diastolic blood pressure; MAP, mean arterial pressure; HR, heart rate.

**Table 2.** Mean Static and Dynamic Post-Extubation NRS and Number of Patients Requiring 0.5 µg kg^−1^ Rescue Fentanyl

	Mean NRS (Static)	Mean NRS (Coughing)	Rescue Fentanyl Consumption (No. of Patients)
At Extubation	2	3.5	4
1 hour	2.1	3.4	Nil
3 hours	2.2	3.4	Nil
6 hours	2.5	3.3	Nil
12 hours	2.5	3.5	3
24 hours	3	4.2	5

NRS, Numeric Rating Scale.
